# Reduced fetal movements

**DOI:** 10.1186/1471-2393-12-S1-A10

**Published:** 2012-08-28

**Authors:** Alexander Heazell

**Affiliations:** 1 University of Manchester, UK

## 

The association between a reduction in fetal movements (RFM) and stillbirth has been noted for at least 450 years. This was formalised from the 1970s onwards in a series of studies that noted the increased incidence of stillbirth and FGR in women presenting with RFM, which in some cases preceded intrauterine fetal death by several days. Interpretation of the literature relating RFM to stillbirth and FGR is complicated by differences in studies’ definitions of RFM and FGR [1]. Nevertheless, the association between RFM and stillbirth remains, irrespective of the definitions used. Recently, the Auckland Stillbirth Study confirmed that women who had a RFM were 2.4 times (95% CI 1.29-4.35) more likely to have a late stillbirth [2], which is strikingly similar to a UK-based study which found a 3-fold increase in stillbirth after one presentation with RFM [3]. RFM, FGR and stillbirth are thought to be related by placental insufficiency, with RFM representing fetal compensation to restriction of nutrients and oxygen *in utero *[4,5]. This hypothesis is supported by evidence of abnormal placental structure and amino acid transport in women with RFM, even in the absence of a small-for-gestational age fetus [6].

Despite the association between RFM and stillbirth, RFM is frequently suboptimally managed clinically. Of 422 stillbirths reviewed in a confidential enquiry, 16.4% of cases had suboptimal care related to RFM, including: not communicating the importance of RFM to mothers and a failure to act on RFM [7]. Reasons for clinicians’ behaviour have been explored by two related questionnaire studies, one in the UK and one in Australia and New Zealand. Both of these studies found significant variations in the definitions of RFM applied to clinical practice and varied knowledge of the association between RFM, FGR and stillbirth. As a consequence clinical management of women with RFM varied significantly, with cardiotocography being used in 80-90% of cases and ultrasound assessment of fetal growth, liquor volume and umbilical artery Doppler in approximately 20% of cases [8,9].

Due to the association between RFM, FGR and stillbirth, ultrasound assessment of fetal growth, liquor volume and umbilical artery Doppler may be useful screening tests to identify placental insufficiency [10]. Norwegian studies have suggested that asking women to be more aware of fetal movements did not increase the number of attendances with RFM. Importantly, the implementation of an associated quality-improvement programme was associated with increased use of ultrasound, but a reduction of stillbirth from 4.2% to 2.4% [11], strongly suggesting that appropriate identification of, and intervention following, RFM may decrease the incidence of stillbirth. The management of RFM may be improved by more sensitive tests to specifically identify placental dysfunction, including measurement of placentally-derived factors such as human placental lactogen or placental growth factor [12,13].

The use of RFM as a screening tool for stillbirth prevention needs to be developed; it has the advantages that it is free and does not significantly increase the burden on the antenatal service. However, the best management protocol after women present with RFM has yet to be determined. To date there have been no randomised controlled trials of the management of RFM, despite calls from the World Health Organisation to improve the quality of evidence regarding stillbirth prevention [14]. Therefore, a high-quality trial is needed to evaluate whether intervention (delivery) directed by appropriate investigations after RFM can reduce the incidence of late stillbirth, without significantly increasing maternal and perinatal morbidity.
